# What are memory-perception interactions for? Implications for action

**DOI:** 10.3389/fpsyg.2014.01553

**Published:** 2015-01-08

**Authors:** Loïc P. Heurley, Laurent P. Ferrier

**Affiliations:** ^1^Laboratory CeRSM EA2931, Center of Research on Sport and Movement, Université Paris Ouest-Nanterre La DéfenseNanterre, France; ^2^Institut Français des Sciences et Technologies des Transports, de l'Aménagement et des Réseaux, Laboratoire de Recherche Mécanismes d'AccidentsSalon de Provence, France; ^3^Laboratory EPSYLON EA4556, Dynamics of Human Abilities and Health Behaviors, Université Montpellier III Paul ValéryMontpellier, France

**Keywords:** embodied cognition, memory, knowledge, perception-action interaction, size perception, grasping movements

Currently, a growing body of studies demonstrates memory-perception interactions (see Barsalou, 2008; Heurley et al., 2012; Lobel, 2014, for reviews). Even if such interactions are highly relevant to support embodied approaches of cognition as well as to better understand memory and perception (e.g., Zwaan, 2008; Versace et al., 2009; Landau et al., 2010; Kiefer and Barsalou, 2013), their functional role remains unclear: Why would perception integrate memory and knowledge while it seems highly efficient without such influences? To understand the functional relevance of these interactions, we assume that it is necessary to take into account two important conditions in which our cognitive systems have evolved during the phylogenesis and continue to evolve during our ontogenesis. More precisely, we develop a view where memory-perception interactions are highly relevant to plan and control actions when we interact with well-known objects in non-optimal perceptual conditions.

It is widely accepted that to properly parameterize action components and to control them during the course of action, it is necessary to perceptually process some object's features (Hommel and Elsner, 2009). As already claimed by Glover (2004), these “action-relevant perceptual features” (ARPF) can be spatial (e.g., shape, orientation) as well as non-spatial (e.g., fragility, weight). Among spatial-ARPF, size is usually recognized as an important cause to its involvement in a great variety of actions. Jeannerod (1984) has for example demonstrated that the magnitude of the grip aperture, a component of the grasping movement, is function to the visual size of objects (see also Ellis et al., 2007; Fagioli et al., 2007; Wykowska et al., 2009). Visual size processing also seems highly important in order to intercept flying objects (Lee, 1976). Nevertheless, very frequently and for various reasons, the perceptual processing of ARPF is far from being optimal especially in “out-of-laboratory” conditions. For instance, some ARPF can't be processed by the available perceptual channels. Indeed, when we want to grasp an object, we are only able to visually perceive it and therefore we are unable to directly perceive its fragility, weight, and temperature whereas they are extremely relevant to plan the force and the velocity of the grasp (Glover, 2004). Even when the right channels are available, some environmental conditions can impair perception. For example, the occlusion of an object by other surfaces can limit our ability to visually perceive it and thus process its shape, size, or distance (Tanaka et al., 2001). Furthermore, short- or long-term injuries to perceptual systems can also induce non-optimal conditions of perception. Indeed, the eyes can be long-term-impaired by cell aging or short-term-impaired by an intense flash light, but in both cases our ability to process visual features is affected. Accordingly, how do we plan and control actions in conditions where features that are suited to plan and control relevant parameters of action cannot often be optimally perceived? First of all, it is important to note that non-optimal processing of ARPF do not necessarily induce object-recognition problems. Indeed, as mentioned in several models, object recognition can be accurately based on non-ARPF such as the color and/or the texture of objects and the context (Tanaka et al., 2001; Bar, 2009). Therefore, even if some ARPF cannot be processed, objects can be accurately identified in many cases. Secondly, because in everyday life we mainly interact with well-known objects, preserved ability to recognize object identity can automatically induce the retrieval of a myriad of knowledge associated with the recognized-objects including associated ARPF (e.g., shape, size). Thus, we claim that recognition processes used to identify objects during the planning phase of actions involve the retrieval of previous experienced ARPF that are automatically integrated into perception. We also claim that they allow compensating non-optimally perceived ARPF and so to maintain a high level of action efficiency even in non-optimal conditions of perception. To resume, we assume that the functional relevance of memory-perception interactions (i.e., an embodied cognitive architecture) occurs when humans interact with well-known objects in degraded-perceptual-conditions. We discuss three potential sets of evidence, coming from studies all focusing on the ARPF size in support of this view.

First, numerous experiments suggests that memory would be able to store objects' perceptual features and especially ARPF (see Barsalou, 2008, for a review). For instance, a great variety of studies support the idea that the size of objects is accurately stored in memory and closely matches their real visual organization (Moyer, 1973; Holyoak, 1977; Holyoak et al., 1979; Shoben and Wilson, 1998; Bertamini et al., 2011; Konkle and Oliva, 2011; Linsen et al., 2011). More importantly, it seems that the known size of objects can be automatically retrieved even when objects are briefly perceived suggesting the possible automatic retrieval of ARPF during fast real interactions with objects. Ferrier et al. (2007) have for example demonstrated that a target picture (e.g., an elephant) is easily categorized as an animal when the brief prime picture (150 ms) has a similar known size (e.g., a giraffe or a car) rather than a different (e.g., a bee or a key) while both pictures have the same visual size on the screen (see also Setti et al., 2009; Gabay et al., 2013). It is noteworthy that the size is generally stable across items of a category as well as across experiences. Because all the ladybugs we experienced have approximately the same small size, their size can be easily stored at a conceptual level (i.e., general knowledge, Whittlesea, 1987). However, in some cases, ARPF could be stored in a more specific or short-term format. For example, because the size of your car is not shared by all the exemplars of the “car” category, this feature is undoubtedly stored in a more autobiographic format. Furthermore, some ARPF are so variable that we can only store them for a short period of time like the last position of your car on the supermarket car park or the distance of some objects on a table (see also Borghi, 2013 for a close distinction). Thus, we claim that ARPF can be stored and automatically retrieved from memory but perhaps in various ways according to the stability of the ARPF across experiences.

Moreover, several studies suggest that ARPF are not only stored but can also influence conscious perception. Among others, the case of the size perception has been strongly studied. In a primary study, Paivio (1975) has demonstrated that the comparison of the known sizes of objects is faster when they are congruent with their visual sizes. In others words, it is easier to say that in general an elephant is larger than a mouse when in the experiment the picture of the elephant is presented larger than the picture of the mouse rather than smaller (see also Srinivas, 1996; Rubinsten and Henik, 2002; Konkle and Oliva, 2012, for similar results). The works of Riou et al. (2011) and Rey et al. (2014) go further and suggest the automatic nature of this influence. Riou et al. (2011) have demonstrated that the known size of objects can influence the detection of a visually odd-sized stimulus in a visual search task while such an object's feature is absolutely useless to complete the task. Others studies have demonstrated an influence of the known size of objects on the judgment of distance that are often derived from visual size suggesting that the stored size can automatically impact not only the perception of visual size but also the perception of other ARPF derived from it (Epstein, 1965; Predebon, 1992, 1994; Hershenson and Samuels, 1999; Distler et al., 2000). Besides the known size of objects, the perception of visual size can be affected by a more abstract kind of size representation: numbers. Henik and Tzelgov (1982) have replicated the interaction between visual- and stored-size reported by Paivio (1975) but with numbers. In a classic bisection task requiring implicit length estimation, de Hevia and Spelke (2009) have found a bias of bisection toward the side of the line where the larger number is printed. In a reproduction task, Viarouge and de Hevia (2013) have demonstrated that large numbers (e.g., 9) presented at each corner of a square induce larger reproduction of this square compared to the condition where smaller numbers are presented (e.g., 2). Altogether, these studies support the possibility that the size stored in memory (i.e., known size of objects or numbers) can directly influence the perception of size or of size-related features (e.g., distance) supporting the possible completion of perception by stored-ARPF when some of them are missing or ambiguous (see Barsalou, 2009, for a similar idea).

A further step is achieved by recent works demonstrating the influence of size stored in memory on more automatic perception-action links (rather than conscious judgments). Indeed, some studies have been able to show an influence of the known size of objects on action parameters that are dependent on the visual size. For instance, Hosking and Crassini (2010) have conducted experiments in which participants have to carry out time-to-contact judgments on stimuli for which a linear or a parabolic trajectory of approach are simulated. Such a judgment is highly important for a great variety of interceptive actions and is mainly based on the online processing of the visual size of the approaching stimulus. In their experiments, the stimuli used have different known sizes (i.e., large: a football vs. small: a tennis ball). Results elegantly demonstrated that this stored feature of objects influences time-to-contact judgments suggesting that it could interfere with our ability to intercept mobiles (see also DeLucia, 2005; Hosking and Crassini, 2011, for similar results). Another set of studies suggest also an influence of the known size of objects on another well-established perception-action link: Our ability to adapt our grip aperture according to the visual size of the to-be-grasped objects (Jeannerod, 1984). Several studies demonstrate that participants are faster to carry out a precision grip on typically small objects (e.g., cherry) and a power grip on typically large objects (i.e., eggplant; Ellis and Tucker, 2000; Tucker and Ellis, 2004; Derbyshire et al., 2006; Girardi et al., 2010) even when visual size cannot interfere (Glover et al., 2004; Tucker and Ellis, 2004; Heurley et al., in revision). The same effect on grip aperture is obtained when size-related adjectives are concomitantly processed (e.g., SMALL/LARGE, LONG/SHORT) rather than known objects (Gentilucci and Gangitano, 1998; Gentilucci et al., 2000; Glover and Dixon, 2002). These results are also replicated when numbers are used. More concretely, Moretto and di Pellegrino (2008) have shown that large number processing facilitate power grips while small number processing facilitate precision grips (see also Andres et al., 2004; Lindemann et al., 2007). In addition, some results support that such interactions are highly automatic (Moretto and di Pellegrino, 2008; Namdar et al., 2014) and seems to be restricted to the planning phase of grasping (Glover and Dixon, 2002; Glover et al., 2004; Badets et al., 2007; Andres et al., 2008). Taken together, these works demonstrate that stored ARPF, such as size, can influence automatic perception-action links and not only conscious perception supporting the possibility that perception can be completed by stored ARPF, itself influencing the planning of some action components.

This short review suggests that the ARPF size can be stored in memory, automatically retrieved during object perception, and can influence conscious perception of visual size (or related-features) as well as the planning of action components mainly based on visual size processing. We used this evidence to support the view that the interactions between present and absent–but simulated in memory–perceptual features are important for action especially in “out-of-laboratory conditions” in which ARPF can't be optimally perceived and in which interactions mainly occur with well-known objects. Of course, the reported evidence is limited to the size, but several studies have already demonstrated that other ARPF such as distance, position, and weight could be stored and automatically retrieved (Estes et al., 2008; Scorolli et al., 2009; Winter and Bergen, 2012). This strongly suggests that our view can be extended. Even if many questions remain open and a lot of work has to be done to best support this view, it has the advantage to search for the functional relevance of memory-perception interactions (i.e., an embodied cognitive architecture) by taking into account two main constraints in which our cognitive systems have certainly evolved at phylogenetic and ontogenetic scales: Interactions with (i) well-known objects in (ii) more or less degraded-perceptual-conditions.

## Conflict of interest statement

The authors declare that the research was conducted in the absence of any commercial or financial relationships that could be construed as a potential conflict of interest.
